# Perceived Causes of Illness Among Infants and Young Children in Bangladesh: An Exploratory Qualitative Study

**DOI:** 10.3390/healthcare13202627

**Published:** 2025-10-20

**Authors:** Md. Fakhar Uddin, Asma-Ul-Husna Sumi, Akash Saha, Mubassira Binte Latif, Shariffah Suraya Syed Jamaludin, Nur Haque Alam, Mohammod Jobayer Chisti

**Affiliations:** 1 Nutrition Research Division, International Centre for Diarrhoeal Disease Research, Bangladesh (icddr,b), G.P.O. Box 128, Dhaka 1000, Bangladesh; asma.sumi@icddrb.org (A.-U.-H.S.); akash.saha@icddrb.org (A.S.); mubassira.latif@icddrb.org (M.B.L.); nhalam@icddrb.org (N.H.A.); chisti@icddrb.org (M.J.C.); 2 Section of Anthropology & Sociology, School of Social Sciences, Universiti Sains Malaysia, Penang 11800, Malaysia; shariffah@usm.my

**Keywords:** perceptions, illness, causes, infants and young children, Bangladesh

## Abstract

Background and objective: Child illness remains a significant public health challenge in low- and middle-income countries, including Bangladesh, with complex multifactorial causes extending beyond biomedical factors. This qualitative study explored perceived causes of child illness from the perspectives of caregivers and healthcare providers in rural and urban Bangladesh. Methods: Twenty-three in-depth interviews with primary caregivers, grandmothers, healthcare providers, and a group discussion with four community representatives revealed four primary categories of perceived illness causes. Results: Individual causes included maternal illness, forgetfulness, and knowledge gaps that affected caregiving practices, leading to missed vaccinations, poor hygiene and feeding practices. Socio-cultural causes included supernatural beliefs, intra-household power dynamics, domestic violence, maternal work burdens, early marriage, adolescent motherhood, and dowry practices. Economic causes included irregular income, rising food prices, and marketing of unhealthy products. Environmental causes included poor housing ventilation, inadequate waste management, heat wave exposure, urban air pollution, and water contamination, causing respiratory and waterborne diseases. Conclusions: These findings illustrate that child illness results from complex interactions between individual, socio-cultural, economic, and environmental causes. Potential interventions can address these multifaceted causes through comprehensive approaches including caregiver education, maternal empowerment strategies, economic support programs, and household environment improvements.

## 1. Introduction

Illnesses are conditions that impair physical, mental, or social well-being, deviating from normal functioning, with symptoms ranging from mild to severe. These include acute conditions such as influenza, bronchitis, tonsillitis, appendicitis, and severe acute respiratory syndrome (SARS) [1]. Child illness remains a major public health challenge in Bangladesh, significantly impacting infant and child mortality rates despite substantial progress in recent decades [2]. The country faces an infant mortality rate of 19.954 deaths per 1000 live births and an under-five mortality of 26 per 1000 live births projected for 2025 [3,4]. In Bangladesh, the under-5 mortality rate of 30 per 1000 live births (2019) remains higher than the South Asian average of 25 per 1000 live births [5,6]. The rate is significantly above the Sustainable Development Goals target of 12 per 1000, placing Bangladesh in the upper tertile among LMICs globally [7,8,9]. Beyond the mortality, widespread morbidity persists, with only 37% of children with fever/cough and 36% with diarrhea receiving medical treatment [10].

Bangladesh, a low- and middle-income country (LMIC), continues battling high rates of preventable childhood illness that affect vulnerable populations, particularly children in urban slums and rural areas with limited healthcare access [11]. In Bangladesh, although diarrhea-related child deaths have decreased, morbidity rates remain high [12,13,14]. Other common child illnesses include malaria (10.3%) [15], dengue fever (12.5%) [16], chikungunya (19%) [17], and typhoid [18,19,20,21]. The burden of child illness in Bangladesh is characterized by a complex interplay of infectious diseases, malnutrition, and inadequate healthcare infrastructure, which collectively contribute to their substantial mortality. This public health challenge not only affects individual families but also poses significant economic and social burdens on communities and the healthcare system. This burden hinders the country’s progress toward achieving sustainable development goals related to child health and well-being.

Current literature in Bangladesh and similar LMIC contexts has predominantly focused on biomedical determinants of child illness such as infectious diseases, malnutrition, and inadequate immunization coverage [22]. Previous epidemiological studies have documented diarrheal diseases, acute respiratory infections, and vaccine-preventable diseases as leading causes of childhood illness in Bangladesh [23]. Quantitative studies have identified multiple risk factors contributing to childhood illness in LMICs, including Bangladesh: poor living conditions, contaminated drinking water, inadequate sanitation and hygiene practices, unhygienic toilet facilities, low parental education, lack of breastfeeding, care-seeking from traditional healers, household sewage proximity, child marriage, cesarean delivery, low family wealth index, and higher numbers of living children in households [24,25,26,27,28]. Research [29,30] has also highlighted the maternal illness, short birth spacing, lack of access to healthcare services, and socioeconomic disparities, with children from lower-income households experiencing higher illness or morbidity rates.

However, existing research has significant limitations. These quantitative studies primarily measure statistical associations between risk factors and child illness outcomes but fail to explore underlying causes from family members’ and healthcare providers’ perspectives. They have limited ability to capture the complex social, cultural, and environmental contexts that influence caregivers’ understanding of illness causation. Furthermore, most research has focused on clinical or biomedical factors while neglecting community perceptions and indigenous knowledge systems that may influence child health outcomes.

Literature suggests categorizing the perceived causes of childhood illness into four categories, including individual, socio-cultural, economic, and environmental causes. Previous qualitative research across LMICs has identified individual causes contributing to child diarrhea, including poor personal hygiene, specific feeding behaviors (such as cow’s milk and formula feeding in infants under six months), and inadequate childcare during maternal absences [31,32,33]. Studies identified some socio-cultural causes, including supernatural beliefs, traditional feeding and childcare practices [32,34]. Economic causes contribute to child illness primarily through malnutrition resulting from food insecurity and delayed or inadequate medical care due to healthcare affordability barriers [35]. Environmental causes of child illness include poor household environment, contaminated water from flooding, open defecation, exposure to extreme weather, and unsanitary living conditions in urban slums [32,33,35,36].

Limited research has examined non-biomedical causes of child illness in-depth in rural and urban Bangladesh from the perspectives of caregivers, family members, and community members. Therefore, we aimed to conduct this qualitative study to explore the perceived causes of child illness in Bangladesh from the perspectives of caregivers, community representatives, and healthcare providers. Understanding these perceptions of illness causation is necessary, as perceived causes influence child recovery, co-morbidity, and care-seeking behaviors. Our study findings can inform culturally appropriate intervention strategies and policies that address the complex pathways and root causes of child illness in Bangladesh. This study finding can also help bridge the gap between community knowledge and formal healthcare systems, contributing to more effective prevention and management strategies for child illness in Bangladesh and similar low- and middle-income country contexts.

## 2. Materials and Methods

### 2.1. Conceptual Framework

**Figure 1:**
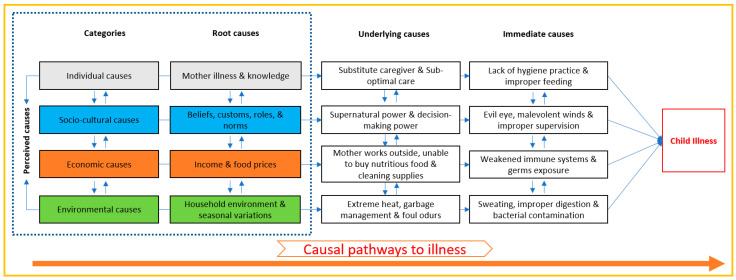
Conceptual framework to explore perceived causes of child illness. Categorized the root causes of child illness into interconnected individual, socio-cultural, economic, and environmental domains based on assumptions. Interconnected immediate and underlying causes of child illness result from these root causes.

We used a conceptual framework, shown in Figure 1, adopted from the UNICEF framework and Uddin et al. 2022 [37,38] to identify the perceived causes of child illness through our empirical qualitative study. Considering the context of Bangladesh, we conceptualized that the root causes of child illness can be broadly grouped into four categories: individual, socio-cultural, economic, and environmental causes. Individual causes can be directly related to the attributes of children, mothers, fathers, grandmothers, and other family members. Similarly, socio-cultural causes can include societal beliefs and customs, while economic causes can include family income and high food prices that contribute to illness. Potential environmental causes can include household conditions and poor adaptation to seasonal variations.

In Figure 1, we placed household environment within the environmental domain (rather than economic) because this cause primarily represents physical living conditions and infrastructure. These conditions directly affect air circulation, room temperature, waste management, and safety—elements we considered fundamentally environmental in nature. However, we acknowledge that household conditions have economic underpinnings and are influenced by economic factors such as income and housing affordability. Similarly, while food costs have economic dimensions, we positioned them based on their more direct relationship to food access and availability, which are also influenced by the food environment. We examined these interconnected causes in this study.

We assumed that these root causes contribute to the underlying and immediate causes of child illness through intersectional causal relationships. Understanding the underlying and root causes behind each immediate cause of child illness is essential for identifying and designing effective interventions to prevent and reduce child illness in the Bangladesh context.

### 2.2. Study Design and Sites

This exploratory qualitative study was conducted between December 2022 and 2025 across rural and urban Bangladesh. The research employed a multi-method qualitative approach that included in-depth interviews and group discussions with diverse participants. We used flexible, semi-structured interview guidelines to enable adaptive questioning during data collection. To enhance validity and reliability, the study implemented triangulation strategies that included multiple data collection methods and coverage of different participant perspectives and contexts.

Data were collected from one urban slum of Dhaka City Corporation and the rural Matlab sub-district in Bangladesh. The study sites were purposively selected to include a range of geographical areas. The rural Matlab, located in the Chandpur District, approximately 55 km southeast of Dhaka, and the urban slums, located in the capital city of Bangladesh. In Dhaka, most of the study participants live in slums, where 37.4% of city dwellers reside in highly contaminated environments [39]. Compared to non-slum urban areas, slum-dwelling children experience higher malnutrition rates, lower immunization coverage, and increased incidence of measles and other infectious diseases. They also suffered more frequently from diarrheal illness and severe dehydration, while their mothers are more likely to work outside the home [40]. On the other hand, rural participants were selected from the Matlab sub-district, which lacks major towns or cities except for the Matlab bazar and has limited inter-village trade and commerce. The population’s dominant occupations are subsistence farming and fishing [41].

### 2.3. Data Collection

We purposively selected twenty ill children from different age groups (0–5 months, 6–11 months, 12–23 months) based on their sex and their mothers’ characteristics (e.g., age, education, occupation). These age categories were selected based on key developmental phases and feeding practices that are important for understanding the causes of child illness. Specific periods include 0–6 months of exclusive breastfeeding (WHO recommendation), start complementary feeding at >6 months, continue complementary feeding up to 11 months while continuing breastfeeding, and transition to family foods with increased environmental exposure from 12–24 months. These age-specific childcares and feeding behaviors may be linked with child illness.

The interviewers went to communities at the selected study sites and visited the community clinics. They took help from healthcare providers at community clinics to identify ill children from the above-mentioned categories. These children were identified from the patient records of the facilities who had received treatment within the last month from the visit day. Following the community clinic’s child list, interviewers visited households to speak with parents and invited them for attending interviews to discuss the perceived causes of their children’s illness.

The mothers and grandmothers of the enrolled children were identified as potential respondents given their likely involvement, experience, and direct or indirect influence on child caregiving practices. Interviewers approached parents of children who had no prior relationship with participants before study commencement, and participants were not familiar with the researchers or interviewers. No one refused to participate in our study. Initially, we planned to recruit a total of 30 participants. However, the final sample size was based on achieving data saturation. We assessed data saturation continuously during data collection in relation to emerging themes linked with study objectives. We achieved thematic saturation when three consecutive interviews yielded no new codes or themes. However, we conducted additional interviews in each group to confirm saturation. The research team met weekly during data collection to assess emerging themes and determine when adequate depth and perspectives had been captured.

Twenty mothers and one influential grandmother of purposively selected children were interviewed to understand their perceptions regarding the causes of their children’s illnesses. We conducted the in-depth interviews (IDIs) separately to minimize the grandmother’s influence on the mothers’ interviews. In addition, we conducted two in-depth interviews with healthcare providers and one group discussion with four community representatives in the communities of the selected children. Interviews and group discussion were conducted at convenient times for the respondents using a flexible semi-structured guideline that was pilot tested. All interviews and the group discussion were audio-recorded, and the average duration of interviews was 48 min.

We selected more samples in urban Dhaka (*n* = 15) than rural Matlab (*n* = 5) based on the epidemiological reality of our study context. Densely populated urban slum areas in Dhaka experience higher childhood illness rates than rural Matlab, due to concentrated air pollution, overcrowding, and poor ventilation.

### 2.4. Data Analysis

All interviews and group discussions were transcribed verbatim. These transcripts helped the study lead (MFU) provide feedback to the other interviewers (AUHS, MBL, and AS). These discussions enabled real-time refinement of the data collection guidelines to capture more comprehensive information in subsequent interviews. We developed a deductive code list derived from the study objectives and data collection protocols for data coding. Following data collection and transcription completion, the research team conducted thorough reviews of transcripts and field notes, applied the deductive code list to four interview transcripts in Microsoft Word, and identified new codes that were integrated into the deductive code list. Finally, this integrated code list was used to code all transcripts using NVivo 14 software. The study lead (MFU) and other team members (AUHS, MBL, and AS) coded each transcript, compared the results, and resolved any discrepancies. We combined a thematic coding approach and a narrative approach in the analysis of data [42], drawing on our conceptual framework (Figure 1) and looking for patterns of similarity and difference across rural and urban areas. Here, thematic coding supplemented and enriched the narrative analysis.

### 2.5. Ethical Approval

The study received approval from the Ethical Review Committee of icddr,b. Prior to conducting interviews, we obtained informed written consent from participants or thumb impressions from those who were unable to read or write. Participants were given assurance that their data would be used exclusively for research and that no names or identifying details would appear in reports or publications. We maintained strict confidentiality protocols to protect all participant information.

## 3. Results

A total of 20 ill children from 20 different households participated in the study. Following their recruitment, we carried out in-depth interviews with 20 primary caregivers and 1 grandmother of a child, conducted 2 individual interviews with healthcare providers, and held a group discussion involving 4 community representatives.

Following the overview of enrolled ill children, participants, and household characteristics, we present the perceived causes of child illness from the perspective of children’s family members, community representatives, and healthcare providers.

### 3.1. Participant and Household Characteristics

The main characteristics of the 20 enrolled children are summarized in Table 1. The ages of the enrolled children ranged from 5 to 23 months, including boys (*n* = 11) and girls (*n* = 9). Most children had various illnesses, such as diarrhea (*n* = 17), fever (*n* = 11), pneumonia (*n* = 4), SAM (*n* = 8), MAM (*n* = 6), jaundice (*n* = 2), cold (*n* = 3), hernia (*n* = 2), rubella (*n* = 2), urinary infection (*n* = 1), and dysentery (*n* = 1). In most cases, the mother of these children was the primary caregiver, who was mostly a housewife (*n* = 17), with one case where the caregiver was the grandmother. Most children lived in extended families (*n* = 12), others in nuclear families (*n* = 8), where the father and grandfather were the main earners and decision-makers. The children came from urban slums in Dhaka city (*n* = 15) and rural areas (*n* = 5).

### 3.2. Perceived Causes of Child Illness

Perceived root causes of child illness are grouped according to our conceptual framework domains, including individual, socio-cultural, economic and environmental. We identified four broad categories and presented study findings and themes under these categories in the results section below.

#### 3.2.1. Individual Causes

##### Maternal Illness

Three respondents (one urban caregiver and two rural caregivers) perceived that maternal illness caused children’s illness by reducing caregiving capacity and breastfeeding practices, depriving children of essential care and nutrients, and resulting in subsequent illness episodes. For example, one caregiver perceived that her child suffered from pneumonia as the child’s mother was hospitalized for gastrointestinal complications necessitating the relocation of the mother along with her sick child from Comilla to Dhaka. During this time, inadequate substitute care and environmental changes contributed to the child developing cold symptoms that progressed to pneumonia. Similarly, an urban caregiver perceived that her postpartum illness led to inappropriate substitute feeding and incorrect medication dosages by a sister-in-law, causing diarrhea in her child. Moreover, a rural caregiver perceived that her heart disease medication reduced breast milk production, forcing formula milk feeding, which resulted in the child developing diarrhea.

##### Forgetfulness and Negligence Towards Care

Four respondents (two caregivers, one child’s grandmother, and one nurse) perceived that caregivers’ forgetfulness and negligence (during busy times with household chores) were primary factors contributing to child illnesses such as fever and diarrhea. These factors led to lapses in preventive care, resulting in inconsistent childcare, missed vaccinations, poor hygiene, and feeding practices. For example, one mother perceived that her younger child experienced persistent fever after the age of six months because of a missed vaccination. She added that while her older child, who had completed the full vaccination, remained healthy.

One nurse mentioned that children experienced recurrent diarrhea and pneumonia after discharge because mothers forgot care instructions and neglected proper feeding, hygiene, food preparation, and medication instructions. She added that mothers’ forgetfulness led to preparing infant formula with incorrect proportions and storing it improperly in containers for extended periods. Additionally, caregivers’ laziness during periods of fatigue resulted in offering previously prepared formula that had been kept at room temperature, demonstrating negligent feeding practices that caused gastrointestinal illness in children.

##### Misperceptions and Knowledge Gaps

Seven respondents (five caregivers, one grandmother, and one nurse) reported that maternal misperceptions and knowledge gaps directly contributed to childhood illnesses. These misperceptions included beliefs that cold-water bathing strengthens children’s immunity, formula milk provides more nutrition compared to breastmilk, and continuous medications cause harmful side effects. These beliefs led caregivers to implement harmful practices, including early antibiotic discontinuation after observing initial improvement, cold-water bathing despite medical advice for using warm water, and inappropriate formula feeding. Healthcare providers subsequently informed these caregivers that their mistaken practices had caused diarrhea, fever, and pneumonia in their children. Three respondents reported that physicians identified premature cessation of antibiotic courses, stemming from caregivers’ fear of medication side effects, as the direct cause of recurrent fever episodes in children.

*“I stopped the medicine after giving it for two days, and the cold problem reduced within three days. I stopped providing medicine as her condition got better, and I don’t like to continue medicine for long. I have a fear that medicine will create more body problems. I don’t take much medicine myself (take one medicine a day, not more than that), and believe that it will be good for the body to have less medicine. But, after stopping the medicine, my child’s cold turned into pneumonia.” IDI-11-Urban*

Five respondents (four caregivers and one nurse) perceived that maternal misperceptions about the nutritional superiority of formula milk over breastmilk led to inappropriate feeding practices for children. This practice caused digestive problems and weakened the immune systems of children, subsequently causing them to become ill, including developing diarrhea.

One nurse stated the following:

*“The child is fed formula milk, but they can’t digest it. Children get attacked by germs, bacteria, and others who don’t get their mother’s breast milk properly. If the child eats any dirt or germs and becomes sick. The child’s body didn’t fight against germs. The child quickly got infected and fell ill.” KII-01-Urban*

Lack of knowledge among caregivers regarding proper hygiene practices directly caused preventable illnesses. One urban caregiver reported that inadequate understanding of feeder cleaning techniques resulted in bacterial contamination that caused her child to experience frequent diarrhea for three months after birth. Her child’s condition only resolved after the caregiver acquired correct cleaning knowledge from her sister, demonstrating how knowledge gaps led to preventable childhood morbidity. One nurse mentioned that caregivers’ lack of awareness about the importance of completion of vaccinations leads to missed immunizations, directly exposing children to preventable diseases.

#### 3.2.2. Socio-Cultural Causes

##### Beliefs in Supernatural Power

Four caregivers believed that the causes of child illness included supernatural forces, including spiritual influences, evil eye (*nazar*), and malevolent winds, leading them to seek traditional healing rather than medical care. These supernatural beliefs influenced caregivers’ understanding of illness causation and treatment approaches. For example, one urban mother expressed that her child had diarrhea and facial swelling due to the evil eye, prompting consultation with a traditional healer who confirmed this supernatural diagnosis.

*“Traditional healer (kabiraj) said that his bowel movement problems were caused by the evil eye or negative energy, which had a negative influence on the house. He also gave an amulet for solving the problems.” IDI-07-Rural*

Another urban caregiver believed that exposure to evil winds in unfamiliar places caused her child’s gastrointestinal symptoms. She explained that after visiting a new location in the evening, malevolent air affected her body and subsequently contaminated her breastmilk, causing her child to vomit and develop diarrhea. These beliefs led her to consistently seek treatment for diarrhea from religious healers at the mosque.

Another caregiver believed that her child had a fever caused by an evil spirit when medical treatment proved ineffective. She subsequently consulted a traditional healer when her child developed measles along with fever, believing that supernatural intervention rather than medical care could lead to her child’s recovery.

*“When my child was small, a young girl visited our home. The girl watched me feed my child, and even after being asked to leave, she continued to observe. Following this, the child’s condition worsened after the feeding, leading to diarrhea. There was a suspicion that the evil air might have played a role, and everyone said that something might be wrong with the girl. The household members believed that the girl’s presence might be connected to the child’s illness.” IDI-07-Rural*

##### Power Dynamics and Decision-Making Power

Three caregivers reported that control of elderly family members in child feeding decisions overrode mothers’ knowledge of safe feeding practices, leading to inappropriate food choices that caused illness. Grandparents’ affectionate but uninformed feeding practices, including providing outside food despite maternal objections, resulted in children experiencing stomachaches and digestive problems. Maternal inability to enforce hygiene standards due to elder authority led to unsafe feeding practices, including serving floor-contaminated food and feeding without hand washing. Elder household members, as supporting caregivers, used traditional feeding methods, such as giving stale milk, which directly caused diarrheal illnesses in children.

*“My child tends to smash rice on the floor and then eat. Grandmother doesn’t perceive that as a problem. Moreover, she (Grandmother) tends not to wash her hands before feeding the child. She doesn’t listen to me and says that she has raised many children for ages and doesn’t need advice. The child’s uncle also fed her food that was brought from outside (Shingara), even though I forbade him. And I told him that the child would get sick eating that food. Other family members do not hear my words (about child caring), though I am the mother. On top of this, I must maintain their (family members’) word about feeding the child. His uncle feeds the child juice twice a day; as a consequence, the child has started experiencing loose stools.” IDI-19-Urban*

Another caregiver mentioned that she was forced to continue formula feeding because her sister-in-law held dominant authority in child feeding decisions, resulting in hospitalization for severe diarrhea at 40 days old. Another caregiver perceived that environmental health risks from nearby sewage, causing her child’s stomach problems, were overruled by her mother-in-law’s refusal to relocate, perpetuating the child’s frequent illness.

*“My in-laws have differing opinions on child care. I visited a renowned doctor and sought medical advice from various sources, but my son’s condition didn’t improve; in fact, it was deteriorating. Due to these circumstances, they told me that I took my child to the doctor unnecessarily, which is why I am hesitant to seek immediate medical attention. However, if my child’s health condition worsens or if the illness proves to be serious, we will have to seek treatment for my child. As my in-laws had different opinions, we faced many challenges when seeking medical treatment.” IDI-14-Rural*

##### Gender Roles and Responsibilities

Overwhelming household responsibilities among child-caring mothers created direct pathways to childhood illness through compromised hygiene practices and inadequate supervision. Five respondents (one nurse, one community representative, and three caregivers) reported that child mothers overwhelmed by household responsibilities struggled to maintain proper hygiene, prepare and feed nutritious meals, and supervise children adequately. Poor hygiene introduces pathogenic contamination, causing infectious diseases and nutritional deficiencies that reduce immunity. Caregivers recognized their divided attention between childcare and household tasks as a direct cause of illness. One caregiver acknowledged that failing to wash her hands before feeding her child due to being busy with other household responsibilities introduced germs through food consumption, resulting in stomach problems.

Prioritizing household chores over child supervision enabled risky behaviors of children, such as playing with contaminated water or mud. Two urban caregivers believed their cold-water exposure during household tasks transmitted illness to their children

*“When I used too much water for domestic work and caught a cold, it might have affected him as well, also tends to get sick. Besides, I can’t always keep the child on my lap; he gets even frequently more fever if he has played on the ground.” IDI-03-Urban*

One rural mother reported her child developed diarrhea after consuming dirty washing water while left unsupervised during household chores.

##### Domestic Violence and Family Conflicts

Three caregivers (mothers) recognized domestic violence and family conflicts as direct triggers for their children’s illness. Verbal and physical violence from husbands and in-laws created acute psychological trauma that diminished mothers’ caregiving capacity, directly resulting in child illness. The emotional trauma from abuse led one mother to develop dependency on sleep medication, which further compromised her ability to provide adequate childcare and subsequently caused her child to become ill.

Two caregivers observed that their children developed fever due to stress-induced immune suppression following incidents where fathers shouted at or physically assaulted the mothers in the child’s presence. One mother described how physical violence against her caused stress-related sleep disturbances and anxiety-induced behavioral changes in her child, including night terrors and fear responses. This disrupted the child’s normal rest and sleep patterns, ultimately leading to the child’s illness.

The child’s father’s physical abuse forced the mother to flee, creating an unstable living situation that disrupted the child’s routine healthcare and increased susceptibility to illness.

Another caregiver shared a case in which the child’s father beat the child’s mother and sent her to the child’s grandmother’s house. After coming to the maternal house, the maternal family was not willing to support her. Because of this, the child’s mother started working in other people’s houses, keeping the child with the maternal grandmother. However, the maternal grandmother was not willing to raise the girl; instead of caring, she kept the two-year-old child on the roadside of the house. Later, the child got diarrhea due to inserting dirty things into her mouth. And, day by day, the girl was getting undernourished due to a lack of proper food.

##### Adolescent Marriage and Motherhood

Three respondents (two caregivers and one nurse) perceived that adolescent marriage led to maternal undernourishment and adolescent pregnancy, resulting in premature and low-birth-weight babies. These babies were prone to respiratory problems, infections, developmental delays, preeclampsia, and anemia. Furthermore, adolescent mothers’ inexperience contributed to improper childcare practices, which directly caused child illness through insufficient breastfeeding and poor hygiene.

One nurse mentioned:

*“Many mothers, being too young and having married early, often lack the necessary knowledge and resources to provide adequate care for their children. These young, inexperienced mothers may not fully understand the details of child care, leading to practices that result in child malnourishment and health issues. Additionally, maternal mental health problems, anxiety, nutritional deficiencies, and lower levels of education further compound the challenges, making it even more difficult for these mothers to ensure their children’s well-being. As a consequence, the children, under the care of such vulnerable and inexperienced mothers, often suffer from malnutrition and various health problems due to the suboptimal caregiving they receive.”KII-01-Urban*

Health workers reported that adolescent mothers faced significant barriers to healthcare access due to several factors, including limited access to household resources, financial constraints, and cultural norms. These barriers restrict their mobility and decision-making power regarding receiving proper childcare and ensuring timely child vaccinations. These releases subsequently increased disease vulnerability and illness progression among their children. Additionally, due to adolescent mothers’ health complications and limited childcare experience, their children often required multiple support caregivers, leading to inconsistent care that further contributes to childhood illnesses.

Repeated pregnancies among adolescent mothers led to preterm birth and low birth weight in children. Short birth intervals (under 18–24 months) cause maternal nutritional depletion, resulting in malnourished infants with weakened immune systems. Moreover, young mothers often experience trauma and depression from early marriage, which impairs their caregiving abilities and negatively affects their children’s physical and emotional development, ultimately leading to preventable childhood illnesses.

#### 3.2.3. Economic Causes

##### Parents’ Low and Irregular Income

Six respondents (two caregivers, one pharmacist, and three community people) perceived that parents’ low- and irregular-income restricted access to nutritious foods and cleaning supplies, forcing them into poor living conditions. These conditions weakened children’s immune systems and exposed them to germs that caused illness. Additionally, fathers’ low- and irregular-income compelled mothers to work overtime to maintain household expenses. This reduced their childcare time and impacted timely feeding and hygiene maintenance, which led to children becoming ill.

One community representative stated that parents’ low and irregular income created situations where families needed 500 Taka for necessities but only had 300 Taka. This forced expense adjustments that compromised nutrition, decreased child immunity, and led to child illness. One community representative stated that,

*“Suppose we need 500 Taka today to buy the necessities, but only have 300 Taka, it creates a shortfall of 200 Taka. This shortage forces us to adjust our expenses and manage within the available 300 Taka. However, this constraint can lead to compromises in nutrition, as we may end up purchasing less nutritious food. If we had the full 500 Taka and could provide better nutrition for our child, I believe that nutrition would not have been compromised, and the child wouldn’t be sick.”GD-01-Urban*

One mother mentioned that her family’s low and irregular income forced them to relocate to a low-cost house with poor hygiene conditions and frequent waterlogging. These conditions contributed to their child’s illnesses, including scabies and diarrhea. Moreover, one nurse mentioned that fathers’ unemployment and low incomes forced mothers to work outside for extended hours, leaving children with inadequate care from inexperienced fathers or siblings, contributing to child illness.

*“When I asked the child’s mother, how does your child get sick? Then the mother replied that I work in garments, and the child’s father stayed at home and looked after our child. But I can’t take proper care of the child. Besides, the child’s father is a male, and can’t be taken care of properly as I(mother) can. But I don’t have any option. I have to work for my family.” KII-01-Urban*

##### Food and Medicine Price Hike

Three respondents (two urban community members and one rural caregiver) reported that food price hikes as a primary driver of illness and its progression among young children. Escalating costs of food and essential medicines prevent families from providing adequate nutrition and timely treatment, directly causing undernutrition, weakened immune systems, and subsequent illness and its progression among children. These factors transform preventable conditions into life-threatening illnesses.

One community representative explained that recent price increases had made vegetables and protein-rich foods financially inaccessible to many families, forcing reliance on cheaper, less nutritious alternatives that compromise children’s nutrition. Additionally, a caregiver reported that medicine price hikes created affordability constraints that delayed medication purchases, allowing her child’s minor cold to progress into serious pneumonia.

##### Commercial Food Marketing and Selling Strategies

Four respondents (two caregivers and two community members) reported that food marketing strategies were the key causes of child illness. Two caregivers explained that local shops strategically place chips, chocolate, and local snacks with appealing packaging, causing children to lose interest in home-prepared meals. Consumption of these foods led to appetite loss and nutritional deficiencies that resulted in children’s low immunity and illness. The low-quality ingredients in these cheap snacks directly caused stomach aches and diarrhea in children.

*“Children often cry for chips and chocolates because they saw them hanging in front of nearby shops. Although we don’t prefer to give them, we unwillingly provide these snacks. Later, the child didn’t want to eat anything and complained of stomach pain, which eventually turned into diarrhea.”IDI-04-Urban*

Two community representatives reported profit-driven pharmaceutical practices as causes of prolonged child suffering and illness progression. They mentioned that some pharmacists persuade parents to purchase specific medicines for profit, regardless of their suitability. Additionally, some drug sellers didn’t sell prescribed medicines but instead suggested purchasing lower-quality and high-profit-margin alternatives, causing disease recurrence and illness progression among children.

##### Contaminated and Low-Quality Food Consumption

Healthcare workers and caregivers reported that excessive chemical fertilizers and pesticides used in food cultivation to increase production and profit margins cause contamination and food poisoning. These chemicals concentrate in fruits, vegetables, dairy products, and meat, leading to illness in children who consume these products, including nausea, vomiting, diarrhea, respiratory problems, and cancer.

The overuse of formalin as a food preservative to prevent financial losses from food spoilage remained a significant concern among study participants. This practice particularly affected vulnerable populations with limited access to fresh, uncontaminated alternatives. One community representative reported that children developed diarrhea after unknowingly consuming formalin-containing fruits purchased from markets. Health workers perceived that formalin exposure through contaminated fruits, vegetables, and fish causes immediate gastrointestinal symptoms in children, including severe diarrhea, vomiting, and abdominal pain. It also increases cancer risk, suppresses immune function, and triggers allergic reactions. Processed foods containing combinations of preservatives, flavor enhancers, and artificial ingredients often cause food allergies and digestive disorders in children.

Economic pressures often forced families to prioritize food affordability and accessibility over safety, leading to purchases from sources with questionable safety standards. Children from low-income families faced disproportionate exposure to food-related contaminants due to limited financial resources that restricted access to organic or certified safe foods. This also forced reliance on cheaper alternatives with potentially higher contaminant levels.

#### 3.2.4. Environmental Causes

##### Poor Household Environment and Waste Management

Two urban caregivers reported poor ventilation, overcrowding, inadequate waste management, and garbage odours in their slum households. They perceived these conditions as causing excessive indoor moisture, contaminated air, disease-carrying flies, children’s excessive sweating from extreme heat, and the development of illnesses from germ exposure.

One caregiver reported her poorly ventilated house lacked natural sunlight and air circulation, creating a moisture-contaminated indoor environment that led to frequent colds in her child and eventually pneumonia. Another urban caregiver mentioned that she had to store solid household waste inside the house because her landlord prohibited outdoor waste disposal. She added that one room beside her living room was used as a storage room for *jorda* (tobacco). The resulting toxic emissions and unsanitary environmental conditions attracted flies and pests, contributing to her child’s gastrointestinal distress, waterborne diseases, and air pollution-related respiratory illness. Another caregiver stated

*“We shared the yard (communal space) with neighbors, and the neighbors left dirty items scattered around the environment. For this reason, the child touches the dirty things and experiences loose motions.” IDI-20-Urban*

##### Extreme Heat Waves

Caregivers perceived extreme summer temperatures and heat exposure as the primary causes of health complications and illnesses in their children. Two respondents reported that prolonged heat exposure deteriorated their children’s health conditions. One caregiver described how her inability to maintain an adequate cooling environment during intense heat periods resulted in excessive sweating that directly caused her child’s pneumonia Another caregiver reported that her child’s heat intolerance directly led to visible physiological distress and the child’s inability to adapt to extreme temperatures. This resulted in skin redness and digestive system breakdown, causing persistent vomiting and diarrhea episodes. Moreover, another caregiver perceived that sudden temperature increases caused recurring respiratory infections and fever episodes in her child that persisted throughout the heat wave period.

##### Air Pollution Exposure

Urban caregivers directly linked their children’s respiratory problems to daily exposure to polluted air in Dhaka from construction activities, traffic emissions, and industrial pollution. They perceived construction dust and particulate matter as major contributors to breathing difficulties and respiratory infections. Community members understood that dust exposure directly causes respiratory diseases if children touch dirty surfaces and fail to maintain proper hygiene. Parents reported that when children breathe polluted air, they immediately develop coughing, rapid breathing, chest tightness, and wheezing. Caregivers recognized winter as the most harmful period when increased air pollution triggers more respiratory symptoms in children, while the monsoon season provides relief with cleaner air and fewer health problems.

*“I used to take her outside every Friday for fresh air and weather, but the outside environment didn’t suit my child; she started nagging, crying, and getting cold due to the dirt. I took her to Gulistan Park in the afternoon when she was 4 months old. Also, the weather was hot, the outside environment was full of dirty things, and the place was crowded (She doesn’t like crowded places, she started crying and saw many people). After coming from there, she got a fever.” IDI-11-Urban*

##### Environmental Contamination of Water Supplies

Participants reported that children become ill in the urban slums of Dhaka due to water supply contamination from multiple sources. Contamination occurs through untreated industrial effluents, improper domestic waste disposal, frequent pipe breaks/leakages, unstructured illegal connections, low water pressure from intermittent service, inadequate sanitation infrastructure, poor domestic water storage structures, and household water-borne food contamination. This widespread contamination makes children particularly vulnerable to waterborne diseases and diarrheal illness.

In overcrowded slums, water contamination is worsened by limited space between water sources and poorly maintained toilets, allowing pathogens to drift from waste facilities to water supplies. The poorest families, lacking resources for consistent protection, remain most vulnerable to childhood illness from contaminated water systems. Community conflicts over shared water sources prevent individual protective measures, and when families attempt mitigation strategies like makeshift scarf filters, social rejection forces them back to consuming contaminated water despite knowing it causes their children’s illnesses.

This creates a cycle where families avoid water treatment until exposure has already sickened their children. Barriers to clean water sources force families to choose between inconvenient access to safe supplies and readily available polluted water containing visible dirt and pollutants. Slum residents identified contaminated water as the primary cause of childhood illnesses, particularly diarrhea and waterborne diseases. One caregiver described how environmental contamination of water supplies harmed her child:

*“My child suffered from diarrhea due to having too much dirt in the supplied water in our area. To solve this problem, I placed a scarf in the tap’s mouth as a makeshift filter. I removed a significant amount of dirt from the scarf after two days of using that scarf. But the other renters disapproved of using a scarf in the tap’s mouth. In response, my mother-in-law provided a clean scarf, but they also disapproved of using it. Consequently, both my child and I continuously remained ill due to drinking water with dirt.” IDI-19-Urban*

Families typically avoided routine boiling until children became ill and struggled with long distances to clean sources. These individual challenges reflect broader systemic problems affecting entire communities, particularly among the poorest families who lacked resources for consistent water purification measures.

##### Perceived Interconnected Causes of Illness

Participants viewed child illness as a complex interconnected web of causation across a range of individual, socio-cultural, economic, and environmental domains. Rather than viewing causes in isolation, they recognized complex interactions between root causes (individual characteristics, socio-cultural norms and beliefs, economic constraints and inequities, and environmental structural factors), underlying causes, and immediate causes (direct exposures and behaviors). This multilayered understanding was particularly developed among caregivers who had witnessed firsthand how causes across different levels mutually reinforced and amplified each other’s effects.

For example, a rural father linked his child’s pneumonia through interconnected causation levels. Root causes included individual maternal health complications compounded by inadequate local healthcare support that necessitated urban hospitalization. These triggered underlying causes, such as forced family relocation and inadequate substitute care arrangements, culminated in immediate causes involving environmental changes and direct exposure risks. Similarly, a rural mother illusively perceived that her heart disease medication (root cause) reduced breast milk (underlying cause), which led to improper formula preparation and feeding practices (immediate cause) that caused the child’s diarrhea.

Moreover, participants’ narratives illustrated how household economic constraints and structural inequalities intersected with socio-cultural marginalization to create poor environmental conditions in households and communities. These conditions included overcrowded living arrangements, shared sanitation facilities, unreliable water systems, and inadequate waste management. These underlying structural causes directly influenced the immediate causes of child illness through contaminated water consumption and compromised hygiene practices. The situation was further exacerbated by significant knowledge gaps and persistent economic constraints that limited household resources available for effective water treatment solutions.

**Figure 2:**
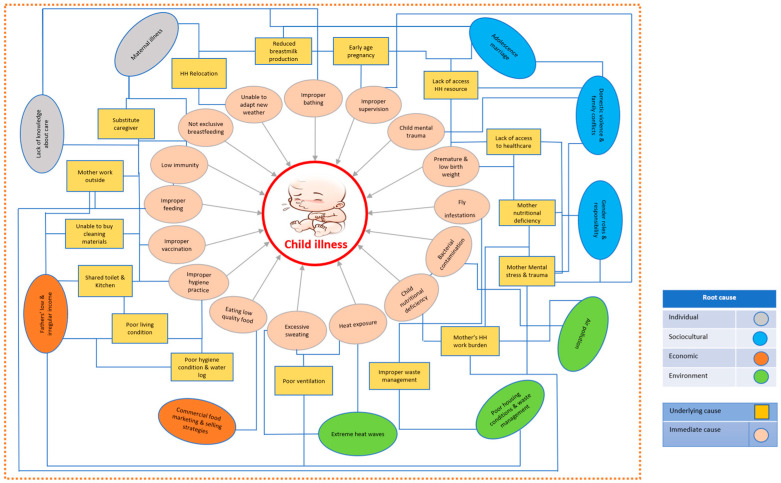
Perceived interconnected causes of child illness.

These examples illustrate participants’ recognition that individual, socio-cultural, economic, and environmental root causes work not as discrete influences but as interconnected forces within complex systems. Here, root causes establish foundational conditions that enable underlying causes to manifest and develop, which subsequently create the circumstances that directly shape immediate causes. Each causal level (root, underlying, and immediate causes) simultaneously influences and receives influence from other levels, creating complex, dynamic networks of causation that continuously interact. Figure 2 below illustrates the comprehensive analysis of perceived interconnected causes of child illness, demonstrating these multilayered relationships and their complex interactions.

## 4. Discussion

This study identified interconnected perceived causes of child illness. These causes fall into four main groups. Individual causes include maternal illness, caregiver forgetfulness, and misperceptions. Socio-cultural causes include supernatural beliefs, domestic violence, and adolescent motherhood. Economic causes include irregular incomes, food price hikes, and predatory marketing. Environmental causes include poor housing, heat exposure, pollution, and contaminated water. Some of these causes are deeply connected to each other. The following paragraphs discuss the intersectional causes of child illness in detail.

Our study uniquely reveals the interconnected pathways between maternal individual causes and socio-cultural-economic causes that create cascading effects leading to child illness. Maternal caregiving knowledge gaps, mental and physical health problems, reduce caregiving capacity, compromise breastfeeding practices and decision-making autonomy, limit economic opportunities, and necessitate care transitions that expose children to inadequate substitute care. Maternal caregiving knowledge gaps were particularly found among adolescent-aged mothers who were married at an early age due to household economic constraints. Mothers’ forgetfulness and negligence during household tasks and family conflicts lead to inadequate hygiene practices during food preparation, poor sanitation practices, and incomplete vaccinations. These disruptions directly contribute to child illness, particularly pneumonia and diarrhea, providing insights that quantitative studies have not fully captured [43]. Our findings partially support previous Bangladesh urban slum research linking maternal mental health disorders to poor child feeding practices that increase malnutrition risk [44,45,46,47,48]. Previous literature showed links between domestic violence exposure and children’s psychological and developmental impairment in Bangladesh [49,50,51]. Our findings show that domestic violence has an impact on maternal mental health that significantly affects their child caregiving capacity, resulting in child illness.

Our research findings suggest comprehensive interventions simultaneously targeting maternal mental and physical health, caregiving knowledge, and structural socio-economic barriers, behavioral factors, rather than addressing individual child illness causes in isolation. Effective interventions can include integrated maternal mental and physical health support programs, community-based caregiving education initiatives, and economic empowerment schemes for mothers. These can address the root causes of the interconnected pathways to child illness. Moreover, a mixed-methods longitudinal study can quantify how maternal illness, caregiver knowledge gaps, and care transitions affect child illness.

We revealed socio-cultural pathways through which caregivers understand and experience illness causation in the urban and rural Bangladesh context. While previous research has documented supernatural beliefs as illness causes across low- and middle-income countries, our findings reveal advanced understanding by demonstrating how supernatural diagnoses impede medical care-seeking, leading to further child illness progression. Moreover, our study findings reveal that the interaction between intergenerational household power dynamics, maternal knowledge, and caregiving inability demonstrates how social structures can override individual protective behaviors. For example, when mothers possess accurate knowledge about contaminated food or water risks, elder family members’ disapproval prevented protective actions by the mother, creating pathways where knowledge alone is insufficient to prevent illness causes. These findings support the previous research in Bangladesh, India, and Ghana, which has shown positive associations between maternal autonomy and child nutritional outcomes [52,53]. Our evidence suggests that effective interventions must move beyond individual maternal behavior change to address the multilayered social determinants identified by communities themselves—including supernatural belief systems, household power structures, and gender-based violence. Moving beyond biomedical and traditional interventions requires culturally informed strategies that acknowledge supernatural beliefs and can address structural inequities in household decision-making power [54].

Our study reveals that families’ economic constraints created a vicious cycle where poverty forces families to make health-compromising purchasing decisions, contaminated foods at cheap rates, despite known risks, which directly causes child illness. In Bangladesh, existing literature has documented widespread food contamination through the use of formalin and excessive pesticides [55,56], parental socioeconomic status, and household wealth as determinants of child health outcomes [57,58,59]. However, our study uncovers previously under-explored economic pathways to child illness, such as community perceptions of food system contamination due to seller economic benefit as a direct cause of child illness, which is often overlooked in quantitative studies. We also explored that when mothers work extended hours due to the household’s economic constraints, childcare quality deteriorates, leading to children’s frequent illness or illness deterioration and increased healthcare costs. This creates a self-reinforcing cycle, particularly devastating for families with irregular incomes. Further research can explore the impact of child illness and its associated healthcare costs on the families of the children.

Our findings extend existing knowledge by revealing how food price hikes and profit-driven junk food marketing strategies specifically targeting children cause child illness and perpetuate the poverty-illness cycle. Interventions can target structural economic inequalities through income support programs, food safety regulations with affordable alternatives, and restrictions on exploitative marketing to children. Families with irregular incomes face unique challenges as they cannot predict or plan for health-related expenses. These households would benefit from flexible income support programs, subsidized healthy food access, and emergency health funding mechanisms that can break the poverty-illness cycle. Future research can evaluate comprehensive poverty-reduction strategies that may address both immediate child health risks and needs and underlying economic vulnerabilities [60].

Our study findings emphasized that family’s economic conditions heavily influenced the living environment of children, their inability to allocate time and materials for maintaining hygiene, which caused child illness. Caregivers reported the environmental root causes of child illness included poor ventilation, creating excessive household moisture and extreme heat, construction dust, and forced indoor garbage storage mandated by the landlord, triggering respiratory symptoms such as pneumonia. Notably, some of these environmental causes interacted with households’ economic constraints, which is reflected in Figure 2 above. Some of these environmental root causes align with previous quantitative research evidence, including poor ventilation increases pneumonia risk in urban Bangladesh children and construction dust significantly contributes to harmful airborne particulates [61,62,63]. A systematic review confirmed that cross-ventilation would be protective against pneumonia in children [64], supporting caregivers’ perceptions about the need for improved ventilation and household waste management infrastructure in urban slums that can prevent environmental causes of child illness. In slum environments specifically, air pollution levels exceed compare to non-slum areas due to industrial proximity, dust from unpaved roads, poor waste disposal, burning of trash, and solid fuels [65,66].

Moreover, while current literature focuses primarily on heatstroke and dehydration [67,68], the caregivers in our study described heat-induced physiological breakdown leading to child respiratory infections through excessive sweating mechanisms not previously captured in quantitative studies [67,68]. Heat exposure interacts with housing quality and economic constraints to create vulnerability where families cannot afford adequate ventilation or cooling measures. These environmental stressors compound existing health risks, particularly for children with pre-existing conditions or undernourished status, creating the risk of illness. Therefore, slum areas with poor ventilation, high construction activity, or limited waste management infrastructure represent priority geographic areas where structural interventions such as improved ventilation systems, cooling and dust control measures, household waste management structure through health investment program and policy could have maximum impact on the reduction in child illness outcomes.

Regarding water contamination, despite accurately identifying contaminated water as causing diarrheal diseases, caregivers revealed concerning social dynamics where community disapproval prevents individual protective measures like filtration, highlighting how collective action barriers can perpetuate exposure to known risks. This challenges conventional water, sanitation and hygiene practices (WASH) interventions that focus on individual behavior change rather than addressing structural determinants and community-level social dynamics that influence health outcomes in slum contexts. We discovered that community disapproval preventing individual protective measures represents a critical gap in WASH literature. Existing research acknowledges that the provision of safe water and adequate sanitation does not always ensure hygienic use or adoption of other sanitary practices [69], but typically attributes this to knowledge gaps rather than social dynamics. Current WASH literature focuses on community mobilization and social capital as positive forces, but lacks documentation of community disapproval as a barrier to individual protective measures [70]. Research in Bangladesh slums showed that “social capital” and community norms were crucial for WASH intervention success [70]. However, studies emphasize “building community ownership of these programs” rather than addressing the negative social pressures that the study identified [69]. Our findings support the growing recognition that structural determinants require different intervention approaches than individual behavior change models [71]. Interventions should prioritize structural improvements, including enhanced ventilation systems and dust control measures in slum housing, while further research should investigate heat-pneumonia pathways and develop community-based approaches that address social barriers to individual protective behaviors rather than relying solely on knowledge-based WASH interventions to reduce environmental causes of illness.

## 5. Strengths and Limitations of the Study

The study used multiple research methods to gather information from different groups of people, including caregivers, grandmothers, healthcare workers, and community leaders from both city slums and rural areas. Obtaining perspectives from many different people and places made the findings more reliable. The researchers used a proven framework to look at causes at individual, social, economic, and environmental levels. They continued collecting data until no new information emerged. The study included poor and marginalized families, which gave important insights into health problems affecting the most vulnerable children. We acknowledge that our small sample size limits the generalizability of our findings. However, this study’s results show the transferability of study findings for a similar population and context, in addition to providing preliminary insights and informing the design of larger-scale research.

The research captured information at only one point in time, so it may have missed seasonal changes or how illness patterns change over time. The study relied on what people remembered and reported, which could be inaccurate. The researchers could not include children’s own views because they were too young to participate. This study was only conducted at two sites due to funding constraints. We distributed the sample unequally and included a small number of samples from the rural site. Future studies would benefit from larger rural samples to enable more robust comparisons between rural and urban populations. Some other study limitations included seasonal variations not captured in our single time-point data collection and limited generalizability beyond the specific geographic regions studied.

## 6. Conclusions

This study explored how child illness in Bangladesh results from a complex interplay of individual, socio-cultural, economic, and environmental causes. Potential interventions can address these multifaceted causes through comprehensive approaches that include caregiver education, maternal empowerment strategies, economic support programs, and household environment improvements. Policymakers should prioritize integrated solutions that tackle the non-biomedical causes of child illness by strengthening existing systems to reduce preventable child illnesses, particularly in vulnerable populations. This study’s findings suggest health investment priorities, emphasizing effective interventions that can address the root causes and other interconnected causes rather than addressing isolated causes.

## Figures and Tables

**Table 1:** Participants and household characteristics.

ID	Children Characteristics					Caregivers/Mothers Characteristics				Households Characteristics			
	Age	Sex	Religion	Illness Since Birth	Caregiver	Age	Education Status	No of Children	Employment Status	Family Type	Earning Person	Income Source	Decision Maker
IDI-01	8 months	Girl	Islam	Diarrhea,	Mother	19 Years	Nine	One	Housewife	Extended	Father	Salesman in a clothing store	Father and mother-in-law
IDI-02	9 months	Girl	Islam	Jaundice, diarrhea, pneumonia	Mother	21 Years	Primary	One	Housewife	Extended	Father	Auto driver	Father
IDI-03	24 months	Boy	Islam	Cold, Fever	Mother	19 Years	Nine	Two	Housewife	Extended	Father	Construction worker	Father
IDI-04	5 months 10 days	Boy	Hindu	Jaundice, pneumonia, convulsions	Mother	27 years	Honors 3rd year	Two	Housewife	Nuclear	Father	Car Driver	Father mother both
IDI-05	17 months	Boy	Islam	Diarrhea, Fever	Grandmother	50 years	One		Sweeper and maid	Extended	Father, grandmother, grandfather	Swiping	Grandmother
IDI-06	9 months	Boy	Islam	Persistent fever, SAM, Diarrhoea	Mother	21 Years	Class nine	Two	Housewife	Extended	Father	Clerk	Father
IDI-07	5 months 19 days	Boy	Islam	Urinary infection, diarrhea, SAM	Mother	26 Years	Class 8	Two	Housewife	Extended	Father	Immigrant	Grandfather, Grandmother
IDI-08	8 Months 21 Days	Girl	Islam	Fever, diarrhea, SAM	Mother	22 years	HSC	Three	Housewife	Extended	Grandfather, Uncle	Tea stall	Father, grandfather
IDI-09	8 months	Boy	Islam	Dysentery, Fever, diarrhea, MAM	Mother	17 Years	Class 8	One	Housewife	Nuclear	Father	Factory worker	Father
IDI-10	6 months 25 days	Boy	Islam	Fever, cold, MAM, and defecation	Mother	24 Years	Class 10	Two	Housewife	Nuclear	Father	Day laborer	Father
IDI-11	6 months 13 days	Girl	Islam	Diarrhea, fever, SAM	Mother	19 years	SSC passed	One	Housewife	Extended	Father, Uncles	Delivery man in Malaysia	Grandfather
IDI-12	11 months	Girl	Islam	Diarrhea, Fever, MAM	Mother,	17 years	Class five	One	Housewife	Extended	Father	Garments worker	Father, Grandfather, Grandmother
IDI-13	7 months	Boy	Islam	Fever, Diarrhea, Rubella, SAM	Mother	21 years	Class five	One	Housewife	Extended	Maternal uncles	Garments worker	Grandmother, Mother
IDI-14	23 months	Boy	Islam	Fever, diarrhea	Mother	25 years	Class five	Two	Housewife	Extended	Father	Construction worker	In-law
IDI-15	21 months	Boy	Islam	Fever, hernia, SAM	Mother	30 years		One	Housewife	Nuclear	Father	Rickshaw driver	Father, mother
IDI-16	10 months 18 days	Girl	Islam	Diarrhea & SAM	Mother	20 years	Class 8 passed	Three	Housewife	Extended	Father	Business (sweet shop)	Father
IDI-17	14 months	Girl	Islam	SAM, diarrhea,	Mother	35 years	Class 9 pass	Three	Housewife	Nuclear	Father	Guard	Father
IDI-18	8 months	Boy	Islam	Pneumonia, diarrhea, rubeola, and hernia	Mother	35 years	Illiterate	Five	Working as a cook	Nuclear	Mother	Cooking	Mother
IDI-19	7 months	Girl	Islam	Pneumonia, cold, and diarrhea, SAM	Mother	19 years	Primary education	One	Worker	Nuclear	Mother	Works in a vest factory	Mother
IDI-20	20 months old	Girl	Islam	Diarrhea, MAM	Mother	20 years	Class 6	One	Housewife	Nuclear	Father	CNG driver	Father

## Data Availability

The data presented in this study are available on request from the corresponding author. The data are not publicly available due to privacy reasons.

## Abbreviations

The following abbreviations are used in this manuscript:

GD	Group discussion
icddr,b	International Centre for Diarrhoeal Disease Research, Bangladesh
IDIs	In-depth interviews
KIIs	Key informant interviews
LMICs	Low- and middle-income countries
SARS	Severe acute respiratory syndrome
WASH	Water, Sanitation and Hygiene
